# Hippocampal Representation of Threat Features and Behavior in a Human Approach–Avoidance Conflict Anxiety Task

**DOI:** 10.1523/JNEUROSCI.2732-19.2020

**Published:** 2020-08-26

**Authors:** Aslan Abivardi, Saurabh Khemka, Dominik R. Bach

**Affiliations:** ^1^Computational Psychiatry Research, Department of Psychiatry Psychotherapy and Psychosomatics, Psychiatric Hospital, University of Zurich, Zurich, 8032, Switzerland; ^2^Zurich, Neuroscience Center Zurich, University of Zurich, Zurich, 8057, Switzerland; ^3^Wellcome Centre for Human Neuroimaging and Max Planck UCL Centre for Computational Psychiatry and Ageing Research, University College London, London, WC1N 3BG, United Kingdom

**Keywords:** anterior hippocampus, decision-making under predation, high-resolution fMRI, hippocampal subfields, lateral amygdala, operant conflict test

## Abstract

Decisions under threat are crucial to survival and require integration of distinct situational features, such as threat probability and magnitude. Recent evidence from human lesion and neuroimaging studies implicated anterior hippocampus (aHC) and amygdala in approach–avoidance decisions under threat, and linked their integrity to cautious behavior. Here we sought to elucidate how threat dimensions and behavior are represented in these structures. Twenty human participants (11 female) completed an approach–avoidance conflict task during high-resolution fMRI. Participants could gather tokens under threat of capture by a virtual predator, which would lead to token loss. Threat probability (predator wake-up rate) and magnitude (amount of token loss) varied on each trial. To disentangle effects of threat features, and ensuing behavior, we performed a multifold parametric analysis. We found that high threat probability and magnitude related to BOLD signal in left aHC/entorhinal cortex. However, BOLD signal in this region was better explained by avoidance behavior than by these threat features. *A priori* ROI analysis confirmed the relation of aHC BOLD response with avoidance. Exploratory subfield analysis revealed that this relation was specific to anterior CA2/3 but not CA1. Left lateral amygdala responded to low and high, but not intermediate, threat probability. Our results suggest that aHC BOLD signal is better explained by avoidance behavior than by threat features in approach–avoidance conflict. Rather than representing threat features in a monotonic manner, it appears that aHC may compute approach–avoidance decisions based on integration of situational threat features represented in other neural structures.

**SIGNIFICANCE STATEMENT** An effective threat anticipation system is crucial to survival across species. Natural threats, however, are diverse and have distinct features. To be able to adapt to different modes of danger, the brain needs to recognize these features, integrate them, and use them to modify behavior. Our results disclose the human anterior hippocampus as a likely arbiter of approach–avoidance decisions harnessing compound environmental information while partially replicating previous findings and blending into recent efforts to illuminate the neural basis of approach–avoidance conflict in humans.

## Introduction

Integrating divergent situational demands is critical to survival, in particular, when predatory or metabolic threat is involved (Korn and Bach, 2015, 2018, 2019). A standard laboratory model of this situation is provided by approach–avoidance conflict (AAC) tests, for example, open-field test and elevated plus-maze (Calhoon and Tye, 2015), which are thought to reflect aspects of human clinical anxiety disorders (Aupperle and Paulus, 2010). Situational threat features are manifold and distinct in these tests, and even more so in biological scenarios (Evans et al., 2019). For a human during wintertime, there is a low probability of being attacked when encountering a hibernating bear and a higher probability when coming across wolves, who are short on food. The metabolic loss incurred by a bear chase, however, may be much higher than when being charged by a single wolf. How the neural system represents and integrates such different threat dimensions, and how they influence behavior (e.g., the decision to approach food under threat or passively avoid threat), remains unknown.

In rodent AAC tests, cautious (“anxiety-like”) behavior is consistently reduced by anxiolytic drugs, such as benzodiazepines (Gray and McNaughton, 2000). Ventral hippocampus lesions have a similar impact (Kjelstrup et al., 2002; Bannerman et al., 2003; McHugh et al., 2004; Ito and Lee, 2016; Kirlic et al., 2017), and it has been suggested that behavioral control requires interplay of hippocampal subfields (Schumacher et al., 2018). Theta oscillations of hippocampal local field potential (Gordon et al., 2005), and synchronization with prefrontal cortex (PFC) (Adhikari et al., 2010; Padilla-Coreano et al., 2016), are increased in AAC, while area-specific circuits influence decisions (Wallis et al., 2019). In a human computer game resembling open-field test, benzodiazepines (Korn et al., 2017) and other anxiolytics (Bach et al., 2018) reduced cautious behavior similar to hippocampus (Bach et al., 2014) and amygdala (Korn et al., 2017) lesions in humans and nonhuman primates (Chudasama et al., 2008; Machado et al., 2009). Amygdala contribution is inconsistently reported in rodents (Kirlic et al., 2017); in humans, it appears to be specifically required for retreat from threat after reward collection, rather than for the decision to approach (Bach et al., 2019).

While this suggests involvement of hippocampus and amygdala in behavioral control, it remains elusive how different threat features, ultimately determining behavior, are represented and integrated. Features, such as magnitude and probability of threat, are not experimentally controlled in many tests that build on innate anxiety or that are extended in time. For example, we have shown using fMRI that neural mass activity of anterior hippocampus (aHC) increases with threat probability in continuous-time AAC (Bach et al., 2014). However, fMRI studies with more abstract AAC tests not requiringimmediate behavior have yielded conflicting results, somesuggesting the same relation of aHC activity with threat probability (Korn and Bach, 2019); others, a relation of aHC activity (Loh et al., 2017) or multivoxel patterns (O'Neil et al., 2015) with behavior.

Operant conflict tests provide the opportunity to moreprecisely control threat features as demonstrated in rodents (Evenden et al., 2009; Oberrauch et al., 2019) and humans (Bach, 2015, 2017; Bach et al., 2019). Here, we capitalized on this latter operant AAC test to disambiguate representation of attack probability, its metabolic cost, and behavior, in aHC and amygdala. We previously used the same task to show that putative hippocampal gamma oscillations, and hippocampal theta synchronization with PFC, increased with threat probability (Khemka et al., 2017). Presently, we gained from the superior spatial resolution of fMRI collecting 1.5 mm isotropic BOLD images focused on amygdala and hippocampus while participants played the game. On each trial, they could either collect, or forego, a monetary token under threat of capture by a predator. Threat probability was defined by the predator wake-up rate and learned by experience; threat magnitude by potential token loss and explicitly signaled.

## Materials and Methods

### 

#### 

##### Participants

Twenty participants were recruited from general and student population in Zurich (mean age ± SD, 23.10 ± 3.34 years; 11 female). Participants had no prior history of neurologic or psychiatric disease and reported normal or corrected-to-normal vision. One participant was excluded from fMRI analysis because of a technical fault in MRI recordings, but included in behavioral analysis. Behavioral results remained consistent after removal of this participant. All participants gave their written informed consent before participation. The study protocol was in full accordance with the Declaration of Helsinki and approved by the governmental ethics committee (Kantonale Ethikkommission Zürich).

##### Experimental procedure

**Figure 1.**
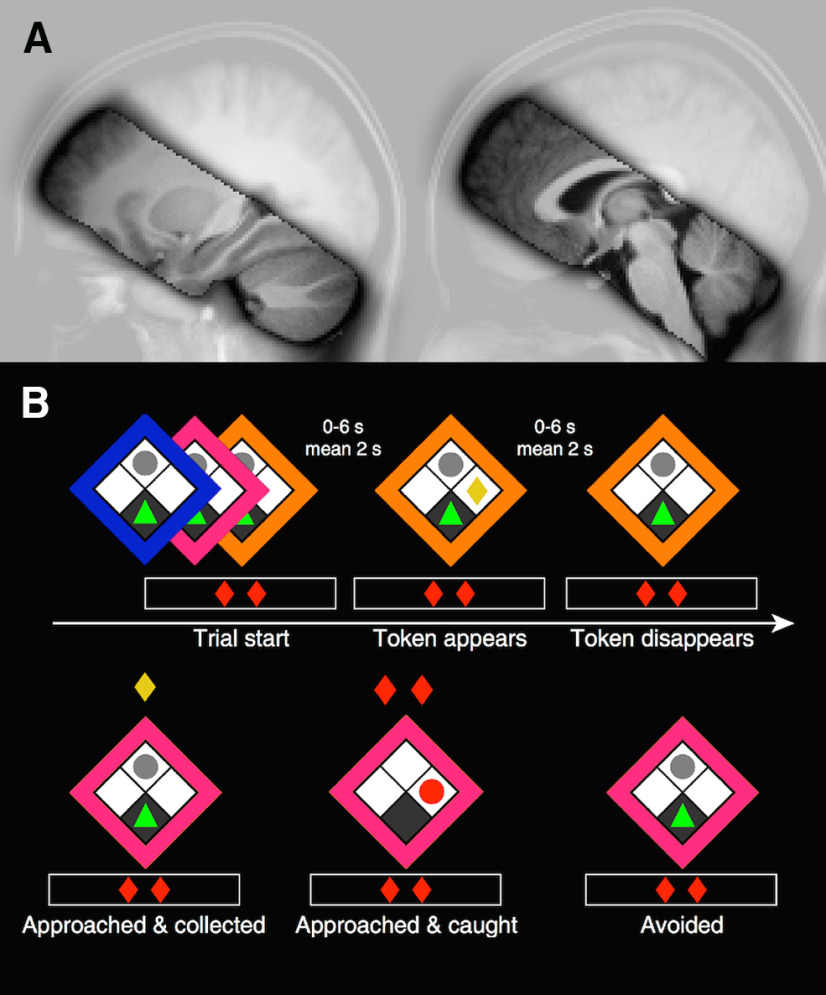
***A***, FOV focused on amygdala/hippocampus. Image represents the EPI coverage across participants (thresholded at *p* = 0.5), overlaid on a mean T1 image in MNI space. ***B***, AAC task. In each trial, the human participant (green triangle) started out in a safe (dark gray) grid block opposite a sleeping predator (gray circle) and was presented with a reward token (yellow rhombus) on the left or right side. Threat probability was signaled by frame color (blue/magenta/orange). The player then had the choice to collect the token using left/right keys to move out of, and return to, the safe place. If caught while outside, the amount of tokens signaled in red below the frame (here two) was lost, thus constituting the magnitude of threat.

Participants performed an AAC computer game as previously used by Khemka et al. (2017), which was modified from Bach (2015). At the beginning of each trial, the human player was located in a “safe place” in the bottom block of a 2 × 2 diamond grid (Fig. 1) opposite of a sleeping predator, and was given the opportunity to collect a monetary token that would appear in the left or right grid block. Red diamonds underneath the grid explicitly signaled the number of tokens that would be lost (0-5) if captured by the predator. Threat probability was implicitly signaled through frame color (blue, pink, and orange). Threat probability was implemented by setting the wakeup rate per time unit to result in catch probabilities of 0.1, 0.2, or 0.3 per 100 ms spent outside of the safe grid block. These probabilities were learned from experience during 36 preceding training trials without token loss that did not count toward ultimate earnings.

A token appeared after a random time interval drawn from a truncated γ distribution (*k* = 2, θ = 1; mean = 2 s, *t* ≤ 6 s). If the player chose not to collect the token, it would disappear after another time interval drawn from the same distribution, and the trial would end 1 s later. If the player went to acquire the token and successfully returned to the safe place, the trial would proceed until the same predetermined end time. Finally, if the predator caught the player, the predator changed its color from gray to red and remained on the screen until the predetermined end time of the trial. After a random intertrial interval also drawn from a γ distribution truncated at *t* ≤ 4 s, during which a blank screen was presented, the next trial would start. Participants completed 648 trials in random order, balanced for each combination of experimental factors (i.e., threat magnitude and threat probability). Participants were instructed beforehand that their payment depended on performance in six trials randomly drawn from the experiment excluding training trials. The experiment was programmed in Cogent (version 2000v1.25; www.vislab.ucl.ac.uk/Cogent) and MATLAB (version 7.14; The MathWorks).

##### Acquisition of MRI data

Data were recorded in a 3.0 Tesla MRI scanner (Phillips Achieva, Phillips Medical Systems) using a 32-channel head coil. Anatomical images were acquired using a 0.76 mm isotropic resolution T1-weighted scan (TR = 7.37 ms, TE = 3.29 ms, flip angle = 8°, FOV = 255 × 255 × 180 mm, matrix = 336 × 336, thickness = 0.76 mm, in-plane resolution = 0.76 × 0.76 mm^2^, slice tilt = 0°, 237 sagittal slices) and a 1.0 × 0.5 × 0.5 mm resolution T2-weighted scan centered on hippocampus (TR = 3200 ms, TE = 353 ms, flip angle = 90°, FOV = 200 × 52 × 200 mm, matrix = 400 × 400, thickness = 1 mm, in-plane resolution = 0.5 × 0.5 mm^2^, slice tilt = 22°, 104 transverse slices). B0 field maps were acquired with a double-echo fast gradient echo sequence (TR = 698.22 ms, TE = 4.10 and 7.10, flip angle = 44°, FOV = 240 × 224 × 240 mm, matrix = 80 × 80, thickness = 3 mm, in-plane resolution = 3 × 3 mm^2^, slice tilt = 0°, 2 × 64 sagittal slices). Functional images during the approach–avoidance paradigm were recorded with 1.5 mm isotropic resolution T2*-weighted EPI sequence (TR = 2800 ms, TE = 30 ms, flip angle = 85°, in-plane resolution = 1.5 × 1.5 mm^2^, FOV = 216 × 54 × 216 mm, matrix = 144 × 144; 36 transverse slices with thickness = 1.5 mm; slice order = interleaved ascending; slice tilt = −40°). FOV was centered on amygdala/hippocampus, but also encompassed striatum, thalamus, prefrontal cortices with exclusion of orbitofrontal cortex and cranio-posterior segments of frontal lobe, greater parts of temporal lobes and cerebellum, as well as complete coverage of insular cortices and brainstem (Fig. 1).

##### Preprocessing of MRI data

Preprocessing of functional images was performed using a standard pipeline in SPM12 (Statistical Parametric Mapping; Wellcome Center for Human Neuroimaging, London; http://www.fil.ion.ucl.ac.uk/spm/software/spm12). In a first step, slice time correction was performed to account for differences in acquisition time of individual brain slices (Sladky et al., 2011). Geometric distortions because of susceptibility-induced field inhomogeneities were addressed using a combined approach, which takes static distortions as well as changes in distortion because of head motion into account (Andersson et al., 2001; Hutton et al., 2002). Static distortions were derived for each subject individually from a B0 field map using the FieldMap toolbox in SPM12. EPIs were subsequently realigned and unwarped integrating the measured static distortion and the estimation of distortion caused by head motion, as well as head motion itself. EPI images as well as T2w images were then coregistered to the individual T1w whole-brain image using a 12-parameter affine transformation. Finally, EPI images were normalized into MNI space and smoothed using an isotropic 8 mm FWHM Gaussian kernel for primary mass-univariate analysis, and a 4 mm FWHM Gaussian kernel for a secondary analysis to improve localization of effects in amygdala and hippocampus. We note that the smoothing kernel must strike a balance between anatomic intersubject variability and regional specificity (Mikl et al., 2008). Thus, the larger smoothing kernel is expected to be more sensitive in detecting activations, but the smaller kernel can provide additional information on the localization of clusters. Unsmoothed EPI images in native space were used for ROI analysis.

##### fMRI analysis (focused brain coverage)

In a primary analysis (P1), we defined a GLM consisting of a δ function at token appearance (consistent with Khemka et al., 2017), convolved with a canonical HRF. Parametric modulators for linear and quadratic effect of threat probability (1-3), linear and quadratic effect of threat magnitude (0-5), and linear interaction effect of threat probability × magnitude were also convolved with the HRF. All parametric modulators were serially orthogonalized. Motion correction parameters were included as six additional regressors of no interest.

To distinguish effects of behavior from threat features, we ran a second parametric analysis (P2) with approach or avoidance as a first parametric modulator, followed by linear and quadratic effect of threat probability, linear and quadratic effect of threat magnitude, linear combination of threat probability × magnitude, linear combinations of approach × probability, approach × magnitude, and finally approach × probability × magnitude.

To extricate effects of threat probability and magnitude that were specific to ensuing behavior, we computed a third GLM with separate trial regressors for approach trials and avoidance trials (P3), each with parametric modulators for linear and quadratic effects of threat probability and magnitude as well as for effect of linear combination of probability × magnitude. To assess a potential relation of neural activity with response latencies, we defined three further models in an analogous manner with parametric regressors for linear and quadratic effects of approach and withdrawal latency during approach trials. We controlled for threat features in all models using serial orthogonalization. Since the player was often caught by the predator during attempts to obtain a token, data for withdrawal latency were available on fewer trials than for approach latency. Thus, we defined one model for approach latency over all trials without control for withdrawal latency, and two models over trials without capture, where approach and withdrawal latencies were orthogonalized in respect to each other.

##### ROI definition

Subcortical and cortical structures, including hippocampus and amygdala, were identified in native subject space using the “recon-all” pipeline in FreeSurfer version 6.0 (http://surfer.nmr.mgh.harvard.edu/) (Dale et al., 1999; Fischl et al., 1999a,b, 2002, 2008; Segonne et al., 2005; Desikan et al., 2006). Individual voxels were assigned neuroanatomical labels in an automated volumetric subcortical parcellation based on a probabilistic atlas from a manual training set (Fischl et al., 2002). The hippocampus segmentation was then further parcellated into anterior and mid-to-posterior hippocampus by automatically splitting the mask at one-third length along the anterior-posterior axis of the image in MATLAB (Strange et al., 2014). For exploratory purposes, CA1 and CA2/3 subfields as well as a mask for dentate gyrus of the were obtained from the higher resolution T2w images with FreeSurfer 6.0, which uses a statistical atlas based on ultra-high-resolution *ex vivo* data images for segmentation. (Iglesias et al., 2015). CA1, CA2/3, and dentate gyrus images were then multiplied with the binary aHC mask to focus only on the anterior segments.

For small-volume correction (SVC) of group-level analysis, a group-level bilateral hippocampus mask was generated by warping the individual bilateral hippocampus masks into MNI space using the deformation fields acquired during normalization of whole-brain T1w images in SPM12. These were then averaged, thresholded at 0.1, and binarized using the SPM12 function ImCalc. For visualization, group-level masks in MNI space for all significant clusters were extracted using SPM12 Results.

##### ROI fMRI analysis

For analysis of estimated condition × condition BOLD response, averaged within ROI, we defined a first-level GLM with separate regressors for 36 possible distinct combinations of threat probability (1-3), magnitude (0-5), and behavioral response (0/1). We extracted estimated condition × condition BOLD response for aHC, anterior subfields CA1 and CA2/3, anterior dentate gyrus, entire amygdala, centrocortical and basolateral amygdala subnucleus groups, and, for visualization, for significant clusters from focused brain analysis.

##### Statistical analysis

Image-based statistical tests for fMRI analysis were performed with SPM group-level analysis using cluster-level familywise error (FWE) correction for multiple comparisons at a voxel-inclusion threshold of *p* < 0.001 (correction for whole FOV, or small volume corrected for hippocampus) and applying a random-field theory based approach as implemented in SPM (Worsley et al., 1992).

For *a priori* ROIs amygdala and hippocampus, we implemented a mixed-effects analysis in R 3.4.3 (www.r-project.org) using function lmer (lme4 package) with the following fixed effects that followed the definition of the voxelwise analysis while adding a hemispheric difference: linear and quadratic effects of threat probability and magnitude, behavioral response and hemisphere, and ensuing interactions. We added a random intercept for subject. This resulted in the R formula (where all predictors are numerical rather than factors):

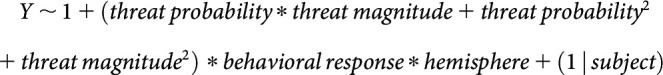


Exploratory analysis in aHC subfields (CA1, CA2/3) and amygdala subnuclei groups (basolateral and centrocortical) was then performed using the same formula. Significance level α was adjusted for multiple comparisons across two ROIs for *a priori* tests, and four ROI for exploratory analysis using the Holm-Bonferroni method (Holm, 1979). To further differentiate for region- and subfield-specific effects in an exploratory analysis, ROI was included as a fixed effect in one combined model for amygdala versus aHC and another for anterior CA1 versus CA2/3. Last, *post hoc* ROI analysis was performed in anterior dentate gyrus using the initial model without ROI as factor. Statistical analysis of behavioral data were likewise performed in R using a linear mixed-effects model (lme4 package), which can deal with the inherently unbalanced data (for details, see Bach, 2015; Khemka et al., 2017), using Satterthwaite approximation to degrees of freedom to appropriately control the false positive rate (Luke, 2017).

##### Data availability

A repository of unthresholded SPM activation maps for parametric analyses P1-P3 (group level; 4 and 8 mm kernel)is publicly available at https://github.com/a-abivardi/neural-threat-behavior-AAC-fMRI (Abivardi et al., 2020).

## Results

### Behavioral results

**Figure 2.**
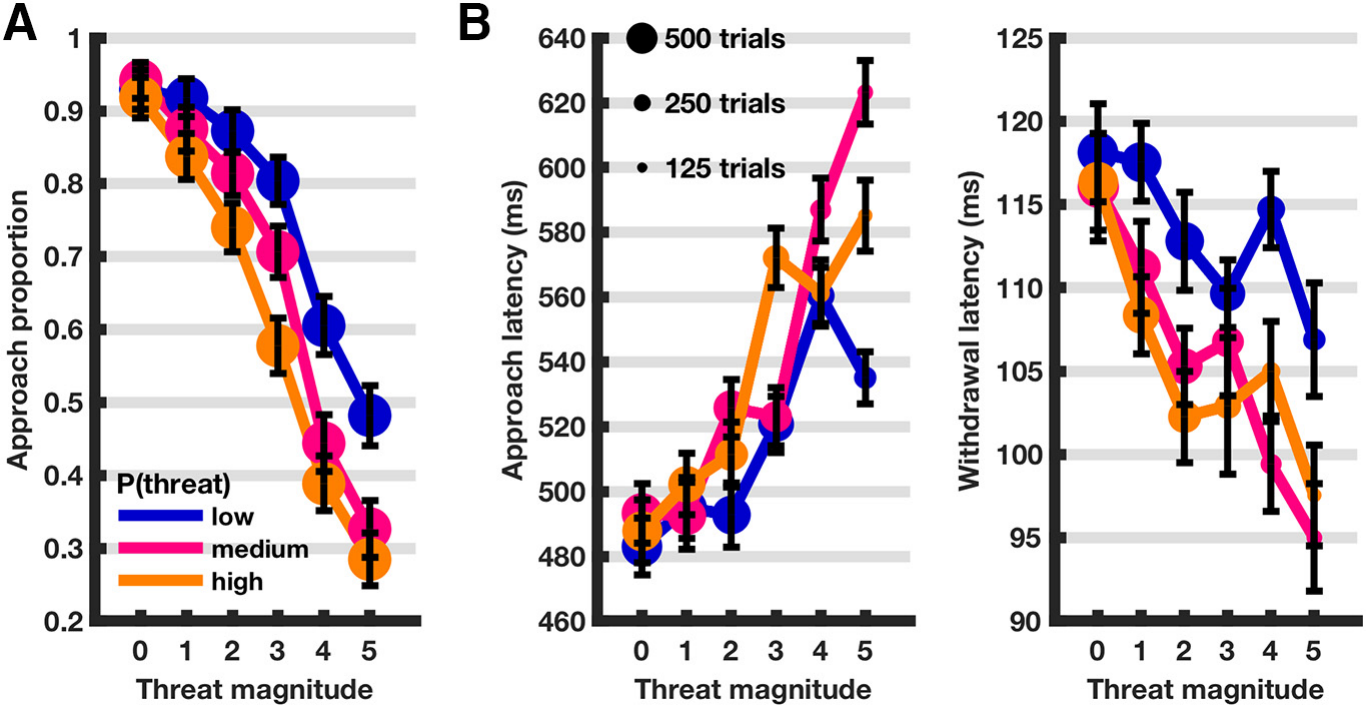
***A***, Proportion of approach–avoidance decisions ± SEM defined as SD of generalized linear mixed-effects model residuals divided by square root of number of data points. ***B***, Approach and withdrawal latency, estimated from linear mixed-effects model ± SEM (defined as SD of model residuals divided by square root of number of data points).

We first interrogated whether behavior was comparable to previous findings. Passive avoidance (i.e., the proportion of avoidance over approach decisions) increased with higher threat probability and magnitude. Behavioral inhibition, measured as approach latency, increased with higher threat probability and magnitude, whereas the opposite pattern was observed for withdrawal latency (Fig. 2; Table 1). These results replicate previous reports (Bach, 2015, 2017; Khemka et al., 2017; Bach et al., 2019) and are in concordance with known behavior from rodent studies.

**Table 1.** Linear and omnibus effects of threat features on behavioral responses

	Action (proportion approach)			Approach latency		
	*F*	df	*P*	*F*	df	*p*
TP: omnibus	61.36	2, 12923.0	<0.001***	12.57	2, 8883.2	<0.001***
TP: linear	214.74	1, 12923.0	<0.001***	14.14	1, 8883.9	<0.001***
TM: omnibus	463.61	5, 12923.0	<0.001***	37.59	5, 8884.8	<0.001***
TM: linear	1945.61	1, 12923.0	<0.001***	173.95	1, 8892.3	<0.001***
TP × TM: omnibus	3.58	10, 12923.0	<0.001***	3.60	10, 8881.6	<0.001***
TP × TM:_linear	15.73	1, 12923.0	<0.001***	3.52	1, 8883.1	0.061
	Withdrawal latency			Movement into correct direction		
TP: omnibus	13.74	2, 7070.0	<0.001***	0.56	2, 8939.0	0.570
TP: linear	18.18	1, 7070.4	<0.001***	1.00	1, 8939.0	0.318
TM: omnibus	10.04	5, 7070.5	<0.001***	1.53	5, 8939.0	0.176
TM: linear	37.90	1, 7074.0	<0.001***	5.57	1, 8939.0	0.018*
TP × TM: omnibus	0.92	10, 7069.1	0.514	0.97	10, 8939.0	0.467
TP × TM: linear	0.80	1, 7069.9	0.371	2.14	1, 8939.0	0.144

Analysis of Variance (ANOVA) of effects of threat features on behavioral measures with Satterthwaite's approximation. Abbreviations: TP = threat probability, TM = threat magnitude.

(*p < 0.05; **p < 0.01; ***p < 0.001).

### Mass-univariate fMRI results

As threat features and approach–avoidance behavior are strongly related, we chose a threefold parametric design (P1–P3) to disentangle distinct effects using serial orthogonalization as implemented in SPM12. In a primary analysis (P1; Table 2), we analyzed how BOLD signal related to linear and quadratic components of the two threat dimensions and their interactions, by including them as parametric modulators. A second analysis (P2; Table 3) prepended these modulators by behavioral response (approach–avoidance), making use of serial orthogonalization in SPM12, and further examined interactions between threat dimensions and behavior. Last (P3; Table 4), approach and avoidance trials were analyzed separately to account for behavior-specific effects of threat dimensions on brain activation. All results were corrected for FWE within the FOV. For bilateral hippocampus, additional FWE SVC was performed using a group-level bihemispheric mask, as we had strong *a priori* hypotheses for this region. Mass-univariate results are reported for images smoothed with an 8 mm Gaussian kernel, unless otherwise specified. Cluster anatomy was visually compared to the schematic brain atlas by Mai et al. (2016). Peak activation coordinates were labeled using the Automated Anatomical Labeling (AAL) atlas (Tzourio-Mazoyer et al., 2002) (Tables 2–4).

**Table 2.** Parametric analysis (P1): effects of threat features on brain activation

Cluster anatomy (manual labeling)	Cluster size	FWE *p* (cluster level)	Peak *z* score	Peak coordinates (MNI; mm)	Peak label (AAL)
Threat probability; negative linear effect					
L middle frontal gyrus (dlPFC)	14	0.049	4.02	−33, 45, 30	Frontal_Mid_L
L putamen; L insula (anterior short gyrus)	17	0.015	3.97; 3.80	−20, 15, −3; −30, 18, −8	Putamen_L; Insula_L
R cerebellum	25	0.015	4.23	44, −47, −33	Cerebelum_Crus1_R
Threat magnitude; negative linear effect					
L anterior limb of internal capsule/putamen	36	<0.001	4.41	−23, 15, 8	Putamen_L
L insula (posterior short gyrus)	16	0.042	4.29	−36, −2, 6	NA
R inferior frontal gyrus, opercular part (vIPFC)	16	0.042	4.28	42, 9, 27	Frontal_Inf_Oper_R
R cerebellum	784	<0.001	4.76	33, −53, −23	Cerebelum_6_R
	82	<0.001	4.50	18, −47, −18	Cerebelum_4_5_R
	56	<0.001	4.30	26, −62, −57	Cerebelum_8_R
	59	<0.001	4.12	9, −72, −26	Cerebelum_6_R
	53	<0.001	4.08	15, −66, −45	Cerebelum_8_R
	34	<0.001	3.92	29, −66, −27	Cerebelum_6_R
L cerebellum	33	<0.001	3.93	−30, −51, −23	Cerebelum_6_L
	16	0.008	3.67	−35, −63, −26	Cerebelum_6_L
Cerebellar vermis	18	0.021	3.76	−3, −59, −32	Vermis_9
L inferior temporal gyrus	17	0.030	3.88	−51, −62, −20	Temporal_Inf_L
Threat probability × magnitude; positive linear effect					
L entorhinal cortex; L presubiculum and parasubiculum extending into CA1 (of aHC)	14	0.043	3.50	−16, −9, −27	ParaHippocampal_L
	12	0.002 (SVC)	3.50; 3.29	−17, −9, −27; −18, −14, −21	ParaHippocampal_L Hippocampus_L

Parametric modulating effects of threat probability, magnitude and their interaction on brain activation (Analysis P1). FWE-corrected results (*p* < .05) at cluster level (whole-brain + whole-brain/small volume corrected (SVC) for hippocampus), at a voxel-inclusion level inclusion threshold of *p* < 0.001. Manual labeling in comparison with schematic brain atlas (Mai et al., 2016). Automated labeling shows AAL (Tzourio-Mazoyer et al., 2002) peak labels verbatim.

**Table 3.** Parametric analysis (P2): effects of approach–avoidance behavior and serially orthogonalized threat features on brain activation

Cluster anatomy (manual labeling)	Cluster size	FWE *p* (cluster)	Peak *z* score	Peak coordinates (MNI; mm)	Peak label (AAL)
Effect of approach					
LR cerebellum	14,178	<0.001	6.74; 6.33; 6.00	14, −63, −52; 18, −50, −21; 27, −56, −20	Cerebelum_8_R; Cerebelum_4_5_R; Cerebelum_6_R
LR ventral anterior, mediodorsal and ventral lateral thalamic nuclei; L ventral posterior lateral thalamic nucleus; LR caudate; L putamen; L insula (posterior short gyrus); L frontal operculum; R habenular nucleus and habenular commissure; periaqueductal gray; R medial geniculate nucleus, L substantia nigra	4677	<0.001	5.50; 5.50; 5.49	−4, −20, 12; −15, −15, 6; 14, −12, 10	Thalamus_L; Thalamus_L; Thalamus_R
R inferior frontal gyrus, opercular part (vIPFC)	220	<0.001	5.03; 4.51; 4.06	58, 15, 0; 62, 14, 14; 57, 10, 8	Frontal_Inf_Oper_R; Frontal_Inf_Oper_R; Frontal_Inf_Oper_R
L substantia nigra	35	<0.001	4.76	−4, −12, −14	NA
LR superior frontal gyrus, medial part (dorsomedial PFC/ACC); LR cingulate gyrus (ACC)	600	<0.001	4.53; 4.51; 4.49	0, 42, 26; 0, 22, 32; −4, 40, 18	Frontal_Sup_Medial_L Cingulum_Mid_L; Cingulum_Ant_L
L cerebellum	40	<0.001	4.41; 3.28	−10, −54, −36; −2, −52, −39	Cerebelum_9_L; Cerebelum_9_L
R insula (anterior short gyrus)	32	<0.001	4.40	42, 8, 2	Insula_R
R inferior frontal gyrus, opercular part (vlPFC); R precentral gyrus	187	<0.001	4.24; 4.20; 4.15	48, 10, 22; 60, 10, 28; 57, 6, 20	Frontal_Inf_Oper_R; Precentral_R; Precentral_R
Effect of avoidance					
L presubiculum and parasubiculum (of aHC)/L entorhinal cortex	10 (SVC)	0.012	3.99; 3.81	−20, −20, −21; −24, −21, −20	ParaHippocampal_L; NA
L anterior CA3/dentate gyrus (of aHC) (4 mm smoothing kernel)	12 (SVC)	0.039	3.66	−21, −18, −18	Hippocampus_L
Threat probability; positive quadratic effect					
L lateral amygdaloid nucleus	25	0.001	4.30	−33, −4, −22	NA
Threat magnitude; positive linear effect					
R insula/area orbitoinsularis (anterior and middle short gyrus); R frontal operculum, R basal operculum	344	<0.001	4.53; 4.39; 4.29	42, 18, −2; 36, 21, −10; 51, 15, −4	Insula_R; Frontal_Inf_Orb_R; NA
L middle hippocampus	11	0.004 (SVC)	4.39	−27, −26, −12	Hippocampus_L
Behavioral response × threat magnitude; positive linear effect (*n* = 16)					
R frontal operculum	21	0.002	3.63	50, 20, −3	Frontal_Inf_Oper_R

Parametric modulating effects of behavioral response (approach/avoidance), followed by threat probability, magnitude and their interactions on brain activation (Analysis P2). FWE-corrected results (*p* < .05) at cluster level (whole-brain + small volume corrected (SVC) for hippocampus), at a voxel-inclusion level inclusion threshold of *p* < 0.001. Manual labeling in comparison with schematic brain atlas (Mai et al., 2016).

**Table 4.** Parametric analysis (P3): effects of threat features on brain activation, separately for approach and for avoidance trials

Cluster anatomy (manual labeling)	Cluster size	FWE *p* (cluster)	Peak *z* score	Peak coordinates (MNI; mm)	Peak label (AAL)
Threat magnitude; positive linear effect (separated approach trials)					
R insula (anterior short gyrus)/R inferior frontal gyrus, opercular part	183	<0.001	4.75; 4.46; 3.66	48, 21, −10; 46, 18, −3; 42, 14, 3	Frontal_Inf_Orb_R; Insula_R; Frontal_Inf_Oper_R
L insula (anterior short gyrus)/L inferior frontal gyrus, opercular part	30	0.001	4.26	−46, 16, −4	Frontal_Inf_Orb_L
	22	0.012	3.90; 3.82	−39, 20, −6; −33, 20, 2	Insula_L Insula_L
LR superior frontal gyrus, medial part (ACC), LR cingulate gyrus (ACC)	158	<0.001	4.25; 4.23; 4.15	0, 27, 28; −2, 34, 26; 6, 38, 24	Cingulum_Ant_L; Cingulum_Ant_L; Cingulum_Ant_R
	39	<0.001	4.06; 3.79	−2, 39, 15; 3, 44, 21	Cingulum_Ant_L; Cingulum_Ant_R
Threat probability × magnitude; linear-positive effect (separated approach trials; *n* = 17)					
L brachium of the inferior colliculus extending into medial geniculate nucleus	19	0.005	4.43	−8, −33, −9	NA
R superior colliculus	20	0.004	4.07	4, −30, −4	NA

Parametric modulating effects of threat probability, magnitude and their interactions on brain activation in separated approach and avoidance trials (Analysis P3). FWE-corrected results (*p* < .05) at cluster level (whole-brain), at a voxel-inclusion level inclusion threshold of *p* < 0.001. Manual labeling in comparison with schematic brain atlas (Mai et al., 2016).

**Figure 3.**
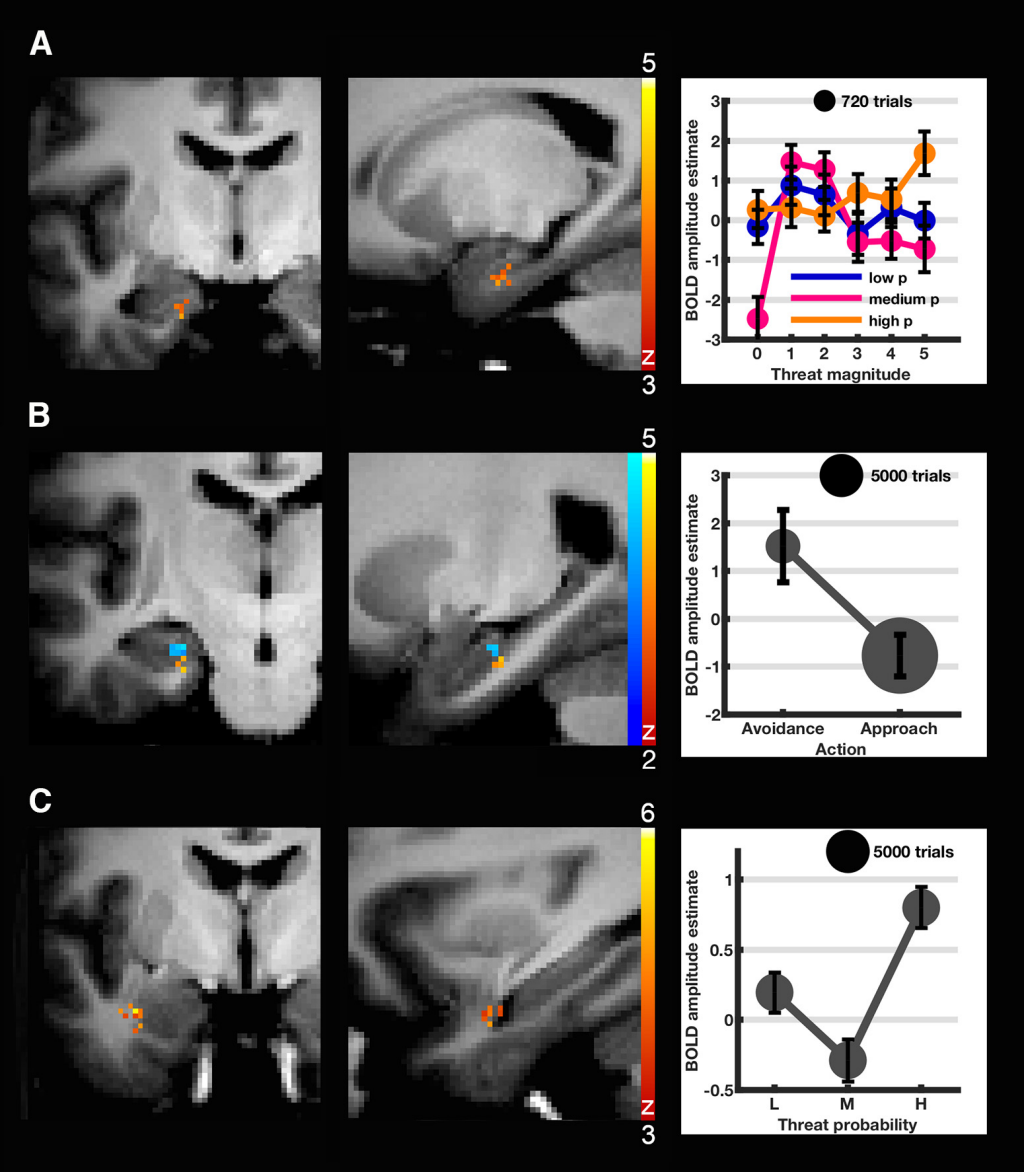
Cluster-level significant aHC and amygdala clusters from parametric analysis with, for purposes of illustration, extracted estimated condition × condition BOLD response ± SEM as defined by SD of BOLD response amplitude estimates divided by square root of number of data points. Red represents primary analysis clusters using 8 mm FWHM smoothing kernel. Blue represents secondary analysis cluster (***B***) using 4 mm kernel. ***A***, Left anterior subiculum-entorhinal cortex cluster modulated by combined threat probability and magnitude (linear-positive interaction effect, analysis P1). ***B***, Left anterior subiculum-entorhinal cortex area relating to avoidance (P2; small-volume corrected). Secondary analysis localized this cluster to the left anterior CA3/dentate gyrus area. BOLD estimates ± SEM are displayed for the 4 mm cluster. ***C***, Left lateral amygdala cluster quadratically modulated by threat probability (P1). All results are FEW-corrected at cluster level (*p* < 0.05; voxel inclusion threshold: *p* < 0.001).

In analysis P1, we observed higher BOLD signal with a combination of higher threat probability and higher threat magnitude in left aHC (specifically subiculum) and entorhinal cortex (linear × linear interaction, FOV-corrected corrected; Fig. 3; Table 2). This effect was not reproduced in a secondary analysis with a narrower smoothing kernel size of 4 mm. There were no mass-univariate effects in the amygdala. Exploratory analysis of the remaining brain coverage (Table 2) revealed higher BOLD signal with lower threat probability (linear negative effect of threat probability) in left dorsolateral PFC (dlPFC), a cluster extending into left putamen and anterior insula, and in the posterior lobe of the right cerebellum. Low threat magnitude was related to higher BOLD signal (linear negative effect of threat magnitude) in left internal capsule/putamen, posterior short gyrus of left insula, ventrolateral PFC (vlPFC), left inferior temporal gyrus, and multiple clusters in bilateral cerebellum and vermis.

In P2, there were no significant hippocampus or amygdala clusters at FOV correction. After SVC in bilateral hippocampus, we observed a cluster in which avoidance behavior related to higher BOLD activity. This cluster in left aHC (subiculum) and entorhinal cortex was located slightly posterior to the area modulated by combined threat magnitude and probability in P1 (Fig. 3; Table 3). This result was replicated in a secondary analysis using 4 mm kernel smoothed images for higher localization accuracy. In this analysis, the cluster was in adjacent location, but more superior in the anterior CA3/dentate gyrus area (Fig. 3). In a distinct cluster in left middle hippocampus (8 mm kernel only), high BOLD signal related to high threat magnitude. Exploratory analysis of the remaining brain coverage (Table 3) revealed that approach behavior related to BOLD signal in two large clusters encompassing bilateral cerebellum and extending from bilateral thalamus to striatum and midbrain structures. Furthermore, approach behavior related to activation in left substantia nigra, bilateral anterior cingulate cortex (ACC) and dorsomedial PFC, anterior short gyrus of right insula, opercular part of right inferior frontal gyrus, and precentral cortex. These clusters showed partial overlap with impact of low threat magnitude as shown in P1, in bilateral cerebellum, left putamen, and anterior insula, as well as with impact of low threat probability in right cerebellum and left putamen. After controlling for behavior in P2, no linear effects of threat probability were observed. A quadratic modulating effect of threat probability emerged in the left lateral amygdaloid nucleus (8 mm kernel only; i.e., high activation for low and for high, but not for medium threat probability) (Fig. 3). High threat magnitude was related to high BOLD signal in right anterior insula (anterior and middle short gyrus) and frontal operculum. A second adjacent cluster in the right frontal operculum showed a linear relation of BOLD signal with threat magnitude specifically in combination with approach (interaction threat magnitude × behavior). This effect, however, was only estimable in 16 subjects.

In P3, there were no hippocampus or amygdala clusters at whole-brain or SVC. Exploratory analysis of the remaining brain coverage (Table 4) revealed that, for approach trials, high threat magnitude was associated with high BOLD signal in bilateral anterior short gyrus of insula, opercular part of inferior frontal gyrus, and bilateral anterior cingulate. Overlap with activation related to approach behavior in P2 was seen primarily in anterior cingulate, whereas overlap with activation related to high threat magnitude in P2 was seen in right anterior insula (replicating the previous finding). Linear interaction of high threat probability and magnitude in approach trials (estimable in 17 subjects) furthermore related to BOLD signal in right superior colliculus (partial overlap with approach-related activation from P2) and a cluster extending from left brachium of inferior colliculus into the medial geniculate nucleus. Specifically, BOLD response increased with threat magnitude for medium and high threat probabilities, but not for low probability.

Effects in avoidance trials were only partially estimable because of unequal distribution (i.e., relative scarcity of avoidance trials across participants) and yielded no significant results.

Finally, we found a positive relation of approach latency with BOLD activation in left ACC and right anterior insula over all approach trials. When controlling for withdrawal latency in the subset of trials where the player was not caught, neither this nor any other relation was seen. There were no significant clusters in relation to withdrawal latency independent of approach latency or threat features.

### ROI analysis results

*A priori* ROI analysis was conducted across both anterior hippocampi, and across both amygdalae. Results were correctedfor multiple comparisons across the two ROIs using Holm-Bonferroni adjusted significance level (Table 5).

**Table 5.** ROI analyses in aHC and amygdala

	*F*	df	*p*
aHC			
A	8.76	1, 1214.6	0.003**
TP^2^	5.46	1, 1208.2	0.020*
TM^2^	14.22	1, 1210.1	<0.001***
TP × H	5.65	1, 1208.0	0.018*
TP^2^ × A	5.50	1, 1208.9	0.019*
TP × TM × H	7.30	1, 1208.0	0.007**
TP × A × H	8.38	1, 1208.0	0.004**
Amygdala
H	8.64	1, 1207.9	0.003*
TP^2^	4.45	1, 1208.2	0.035*
Anterior CA1
TP^2^ × A	8.88	1, 1208.8	0.003**
TP × TM × H	8.65	1, 1208.8	0.003**
Anterior CA2/3
A	10.14	1, 1222.6	0.001**
Basolateral amygdala
TM	6.26	1, 1210.9	0.012*
H	11.13	1, 1207.5	<0.001***
Centrocortical amygdala
H	6.81	1, 1208.1	0.009**
TM^2^	9.28	1, 1208.4	0.002**
TM^2^ × A	6.77	1, 1208.1	0.009**
Combined model: aHC + amygdala
A	9.91	1, 2434.9	0.002**
ROI	13.61	1, 2433.9	<0.001***
TM^2^	10.23	1, 2434.2	0.001**
TP × H	6.05	1, 2433.9	0.014*
A × ROI	7.87	1, 2433.9	0.005**
TM^2^ × ROI	4.98	1, 2433.9	0.026*
TP × TM × H	6.82	1, 2433.9	0.009**
TP × A × H	8.50	1, 2433.9	0.004**
TP × H × ROI	4.39	1, 2433.9	0.036*
TP × TM × H × ROI	4.37	1, 2433.9	0.037*
TP × A × H × ROI	5.87	1, 2433.9	0.015*
Combined model: anterior CA1 + anterior CA2/3
A × ROI	4.95	1, 2670	0.026*

Main and interaction effects significant after Holm-Bonferroni correction (*p < 0.05; **p < 0.01; ***p < 0.001) from Analysis of Variance (ANOVA) of mixed effects model of estimated condition-by-condition BOLD response averaged across region-of-interest (entire/subregional amygdala and anterior hippocampus). Significant main and interaction effects from ANOVA of combined mixed effects model for amygdala vs. anterior hippocampus and anterior CA1 vs. anterior C2/3. Abbreviations: TP = threat probability, TM = threat magnitude, A = action, H = hemisphere, ROI = region-of-interest.

**Figure 4.**
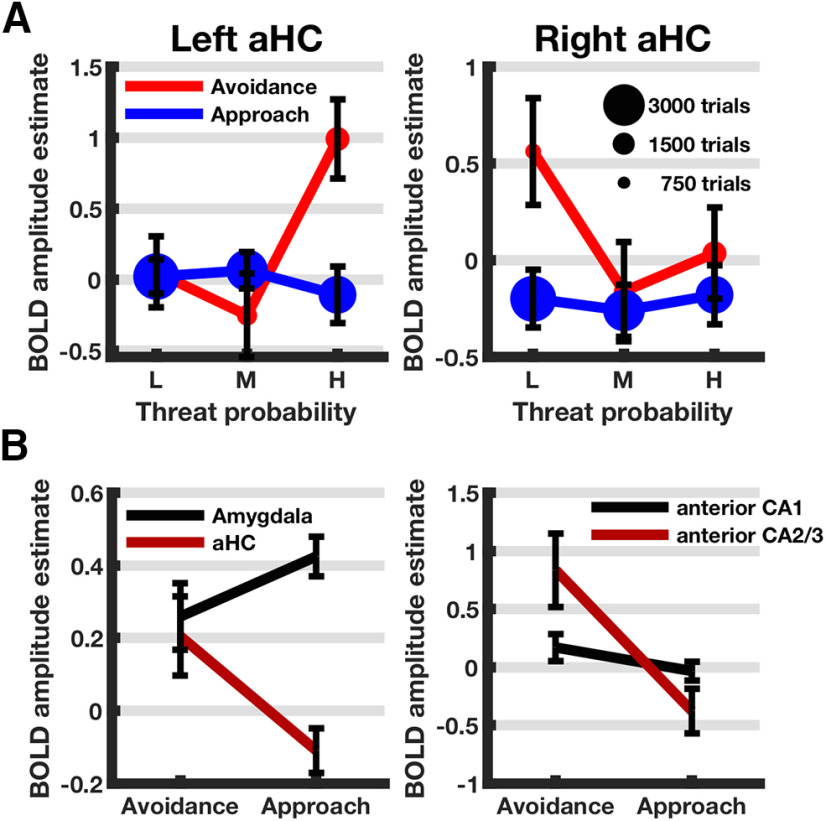
ROI analyses for aHC (***A***), amygdala versus aHC (***B***), and anterior CA1 versus anterior CA2/3 (***B***). ***A***, Interaction effect of threat probability, approach, and hemisphere: that is, estimated condition × condition BOLD response amplitudes ± SEM defined as SD of mixed-effects model residuals divided by square root of number of data points. ***B***, Interaction effect of behavior × ROI for amygdala versus aHC and anterior CA1 versus anterior CA2/3 (condition × condition BOLD response ± SEM).

In the aHC ROI, we observed a linear main effect of behavioral response and quadratic main effects for threat probability and magnitude. BOLD signal was higher for avoidance than for approach trials. Similar to left lateral amygdala in parametric analysis P2, aHC also responded to low and high threat probability (positive quadratic effect). Strikingly, this effect seemed to be behavior-dependent and lateralized as left aHC responded to high threat probability and right hippocampus activation related to low threat probability, both during avoidance only (quadratic × linear interaction of threat probability and behavior, and linear interaction of threat probability × behavior × hemisphere) (Fig. 4). Moreover, for zero threat magnitude, hippocampus BOLD signal was low, while increasing to peak levels for low to intermediate levels and falling again with higher magnitude, resulting in a significant negative quadratic pattern. Finally, aHC exhibited a complex linear interaction of threat features and hemisphere: BOLD response showed a negative linear relation with threat magnitude for high threat probability and for left hemisphere only.

The response to low and high threat probability seen in lateral amygdala after control for behavior was replicated in the amygdala ROI analysis (positive quadratic main effect), while interactions between threat probability and behavior were not detected. Left hemisphere showed overall higher BOLD responses in the amygdala.

A combined analysis of amygdala and aHC revealed distinct activation patterns in relation to behavior (Fig. 4; Table 5). While aHC was clearly more active during avoidant behavior, amygdala exhibited a slightly higher BOLD response during approach (behavior × ROI interaction). The quadratic response to threat magnitude appeared to be specific to aHC; moreover, aHC was different from amygdala in its lateralized response to threat probability during avoidance (linear threat probability × magnitude × behavior × ROI interaction).

In addition to planned ROI analysis, exploratory follow-up analyses were conducted in bilateral aHC subfields CA1 and combined CA2/3 as well as basolateral and centrocortical amygdala ROIs. Results were corrected for multiple comparisons across four ROIs using Holm-Bonferroni method (Table 5).

Subfield analysis in anterior CA1 revealed complex and interacting effects of threat dimensions with distinct activation patterns for approach and avoidant behavior and depending on hemisphere. As in entire aHC, a relation to low and high threat probabilities was seen during avoidance only (quadratic × linear interaction). A complex linear interaction effect of threat features and hemisphere also similar to entire aHC was observed.

Activation in combined hippocampal subfield CA2/3 was higher for avoidance than approach behavior, reflecting the main effect found in the combined aHC ROI. The difference (or increase) in BOLD response for avoidance compared with approach conditions was furthermore higher for anterior CA2/3 (mean = 1.31, SD = 1.28) than for subfield CA1 (mean = 0.22, SD = 1.17) in a *post hoc* paired sample *t* test (*t*_(17)_ = −3.31, *p* = 0.004), underlining the difference between the two subfields. A combined model for the subfields confirmed this distinction with a significant behavior × ROI interaction effect (Fig. 4; Table 5). At the suggestion of a reviewer, we analyzed BOLD responses in anterior dentate gyrus, based on findings that this area may have a role similar to that of CA3. However, we did not find a significant relation with avoidant behavior here.

Further exploratory analysis in amygdala subnuclei using a probabilistic amygdala mask from a previous study (Abivardi and Bach, 2017) revealed activation of basolateral amygdala with increasing threat magnitude (linear main effect). This effect was not seen for entire amygdala. Left basolateral and centrocortical amygdala were more active than amygdala of the right hemisphere as also seen for entire amygdala. Also, centrocortical amygdala exhibited heightened BOLD response to intermediate threat magnitudes, especially during avoidance (quadratic main effect + quadratic × linear interaction). We note that the centrocortical amygdala parcellation was defined by structural connectivity with lateral orbitofrontal cortex (Bach et al., 2011) based on preferred projections to central, medial, and cortical amygdala in rodents and nonhuman primates (Carmichael and Price, 1995; McDonald, 1998; Pitkänen, 2000). Morphologically, this group parcellation (resulting from a sample with size of *n* = 50) (Abivardi and Bach, 2017) probably includes central, medial, cortical, as well as basomedial nuclei.

## Discussion

Rodent and human ventral or aHC is crucial to cautious behavior in AAC tests (Ito and Lee, 2016). However, how distinct threat features are represented and integrated has only recently received attention (Korn and Bach, 2019). Harnessing a human operant AAC computer game during high-resolution fMRI, we investigated representation of threat probability and threat magnitude, and of approach or avoidance behavior, in aHC and amygdala. Two key findings emerged. First, aHC BOLD activity was related to behavioral avoidance, particularly for CA2/3 but not for CA1. Second, there was no evidence that aHC unambiguously represents elementary threat features in a linear manner. Similarly, exploratory analyses of further brain areas within our limited coverage did not reveal a coherent linear representation of threat probability or magnitude.

In mass-univariate analysis, we observed that BOLD signal in left aHC/entorhinal cortex, specifically the subiculum-entorhinal area, was related to the combination of high probability and magnitude of threat (analysis P1), both of which result in more avoidant behavior. After controlling for behavior (P2), no such relation was found. Instead, neural activity in a slightly more posterior cluster was related to avoidant behavior. Using a smaller smoothing kernel to fully harness high spatial resolution, we localized this second cluster to the anterior CA3/dentate gyrus area. *A priori* ROI analysis confirmed these findings:averaged aHC BOLD signal was increased during avoidance. Follow-up analysis of anterior subfields revealed that this avoidance-related increase occurred in CA2/3 but not CA1. This finding resonates with a rat experiment by Schumacher et al. (2018) who demonstrated that selective pharmacological inactivation of ventral CA3 increased approach behavior. A role paralleling CA3 has been recently described for rodent ventral dentate gyrus (Yeates et al., 2020). We note that it remains possible that our CA2/3 parcellations contain individual voxels belonging to bordering dentate gyrus. Nonetheless, exploratory ROI analysis in dentate gyrus did not detect a similar effect here. On the other hand, CA1 activity in our study showed no simple relationship with threat features or behavior, whereas selective pharmacological ventral CA1 inactivation increased avoidance in a previous rat experiment (Schumacher et al., 2018).

Our finding of aHC activity relating to avoidance is in keeping with a previous human fMRI study involving abstract AAC decisions, which reported inferior aHC BOLD activity during avoidance (Loh et al., 2017), in proximity to the left aHC cluster relating to avoidance here. We note that, in this previous study, most voxels in this cluster were labeled as belonging to CA1; however, the authors noted that anatomic specificity might have been limited because of lower spatial resolution (3 mm), as opposed to the present approach.

In a lesion study with the same paradigm as used here, we found that hippocampus lesions impaired approach–avoidance decisions, whereas impact of threat on other behaviors remained intact (Bach et al., 2019), further suggesting a specific role of aHC in generating avoidance behavior. Selective amygdala and hippocampus lesions were moreover associated with shorter approach latency, but not with a different relationship between threat and approach latency. This may suggest that these regions do not contribute to parametric variation in approach latency. In keeping with this, we presently found that variation in approach latency did not relate to hippocampus or amygdala signal. A previous magnetoencephalography study reported a relation between approach latency and posterior hippocampus activity (Khemka et al., 2017), not observed here.

Regarding threat feature representation, ROI analysis revealed a more complicated picture than previously assumed. Although we observed significant responses of aHC to low and intermediate threat magnitude levels, forming a quadratic pattern, BOLD signal also depended on interactions between threat features and behavior, with some effects strikingly different between hemispheres. Specifically, left aHC responded to high threat probability, whereas right aHC related to low probability during avoidance. In humans, left hippocampus has been implicatedin contextual and spatial memory encoding, whereas right hippocampus has been linked to navigation accuracy (Maguire et al., 1998; Spiers et al., 2001). Hemisphere-specific connectivity profiles in human aHC (Robinson et al., 2016) and task-related activity in rat ventral HC (Sakaguchi and Sakurai, 2017) have been reported. However, we note the historical and ongoing debate on lateralization of emotional functions, which is based on partly contradicting observations (Gainotti, 2019). It would therefore appear useful to replicate our findings in an independent sample.

In contrast, a previous fMRI study (Bach et al., 2014) using a more ethological paradigm reported linearly increasing activity in left aHC with higher threat probability. Accounting for the influence of behavior in this temporally extended paradigm was, however, difficult. Furthermore, previous threat probabilities were higher (0.2/0.5/0.8) than the current ones (0.1/0.2/0.3). Also, this previous study did not explicitly control threat magnitude, which we achieved here. Another fMRI study involving more abstract foraging decisions under predation (Korn and Bach, 2019) found a cluster in which aHC signal increased with threat probability (0.1–0.4) but a partly overlapping cluster in which aHC signal decreased from 0.1 to 0.3 and increased from 0.3 to 0.4, yielding an overall quadratic pattern. To reconcile these findings, it appears necessary to cover a larger probability range.

As a further finding, BOLD signal in left lateral amygdala related to low and high, but not intermediate, threat probability independent of behavior (P2). ROI analysis in entire amygdala replicated this behavior-independent activation pattern. The role of amygdala in AAC is reported more controversially than for aHC (Kirlic et al., 2017); nevertheless, a recent human lesion study suggested specific involvement in controlling vigor of return to safety (Bach et al., 2019).

Results from exploratory focused brain analysis revealed several clusters with complex and differential relation with threat features and behavior. Left dlPFC response was related to low threat probability (P1), resonating with reports that anxiety is inversely correlated with dlPFC activity (Balderston et al., 2017). Right vlPFC has been implicated in motor inhibition and characterized as a “brake,” which however has been debated (Aron et al., 2004, 2014; Swick and Chatham, 2014). Here, right vlPFC activity related to low threat magnitude (P1), approach behavior (P2), and high magnitude during approach (P3). While we did observe behavioral inhibition during approach trials relating to threat, the relation to approach behavior seems at odds with pure motor inhibition. Swick and Chatham (2014) propose that vlPFC monitors action-relevant situational changes, compatible with response to threat magnitude here.

In a recent optogenetic study, ACC activation decreased rodent freezing behavior via input to basolateral amygdala (Jhang et al., 2018). Anterior cingulate also appears to signal value predictions of rewards and punishments (Monosov, 2017). Conceptually, dorsal anterior cingulate has been theorized to monitor conflict (or expected value of top-down control) (Botvinick et al., 1999; Shenhav et al., 2016) or to adaptively track context-relevant and action-guiding variables (Heilbronner and Hayden, 2016). Here, dorsal anterior cingulate related to approach behavior (P2) while also relating to rises in threat magnitude during approach trials (P3). The former finding matches anterior cingulate role in freezing in mice and supports a more active role arbitrating behavior. The latter finding may equally well constitute measurement of conflict, context-relevant variable tracking or punishment-related value predictions.

Anterior insular cortex activity was related to approach decisions and both threat features. Left insula related to low threat magnitude and probability before accounting for behavior (P1), right anterior insula activation was related to approach (P2) and bilateral insula to high threat magnitude in separated approach trials (P3). This contrasts reports from a study implicating anterior insula activation in avoidance decisions (Aupperle et al., 2015). Overall, insula showed similar responses to anterior cingulate, adding to evidence of their close functional link (Medford and Critchley, 2010).

Limitations of our study include the use of a limited FOV as a necessary compromise for higher-resolution imaging of ROIs. Furthermore, a relative scarcity of avoidance decisions across participants, compared with previous studies with cumulative token collection (Bach, 2015, 2017; Bach et al., 2019) hindered analysis of threat representation during avoidance and reduced power to detect brain areas involved in avoidant decision-making. A focus on single-stage decisions in the present study precludes analyzing to what extent assumptions about future foraging attempts may prompt avoidance on the current one (Korn and Bach, 2019; Zorowitz et al., 2020). Last, orthogonalization in SPM12 penalizes parametric modulators in a serial manner along the design matrix, which demands careful interpretation of results (Mumford et al., 2015).

In conclusion, in this study, we disambiguated a relation of neural tissue activity with behavior and situational threat features. AHC BOLD signal, in particular in CA2/3, increased when participants avoided threat. Representation of threat features showed a complicated pattern, and for threat probability depended on behavior. This is in line with a notion that hippocampus does not linearly represent threat features but retrieves them, possibly in a manner that changes over time, to compute decisions. It would be useful to increase the range of these threat features, as well as improve both spatial and temporal precision of recording, for example using electrophysiology, to understand how these computations emerge over time in different hippocampal subfields.
